# Availability of drugs and medical supplies for emergency obstetric care: experience of health facility managers in a rural District of Tanzania

**DOI:** 10.1186/1471-2393-14-108

**Published:** 2014-03-19

**Authors:** Dickson Ally Mkoka, Isabel Goicolea, Angwara Kiwara, Mughwira Mwangu, Anna-Karin Hurtig

**Affiliations:** 1 Department of Clinical Nursing, School of Nursing, Muhimbili University of Health and Allied Sciences, Dar es Salaam, Tanzania; 2 Department of Development Studies, School of Public Health and Social Sciences, Muhimbili University of Health and Allied Sciences, Dar es Salaam, Tanzania; 3 Department of Public Health and Clinical Medicine, Unit of Epidemiology and Global Health, Umeå University, Umeå 901 85, Sweden

**Keywords:** Health facility managers, Health system governance, Emergency obstetric care, Integrated logistic system, Medical store department, Tanzania

## Abstract

**Background:**

Provision of quality emergency obstetric care relies upon the presence of skilled health attendants working in an environment where drugs and medical supplies are available when needed and in adequate quantity and of assured quality. This study aimed to describe the experience of rural health facility managers in ensuring the timely availability of drugs and medical supplies for emergency obstetric care (EmOC).

**Methods:**

In-depth interviews were conducted with a total of 17 health facility managers: 14 from dispensaries and three from health centers. Two members of the Council Health Management Team and one member of the Council Health Service Board were also interviewed. A survey of health facilities was conducted to supplement the data. All the materials were analysed using a qualitative thematic analysis approach.

**Results:**

Participants reported on the unreliability of obtaining drugs and medical supplies for EmOC; this was supported by the absence of essential items observed during the facility survey. The unreliability of obtaining drugs and medical supplies was reported to result in the provision of untimely and suboptimal EmOC services. An insufficient budget for drugs from central government, lack of accountability within the supply system and a bureaucratic process of accessing the locally mobilized drug fund were reported to contribute to the current situation.

**Conclusion:**

The unreliability of obtaining drugs and medical supplies compromises the timely provision of quality EmOC. Multiple approaches should be used to address challenges within the health system that prevent access to essential drugs and supplies for maternal health. There should be a special focus on improving the governance of the drug delivery system so that it promotes the accountability of key players, transparency in the handling of information and drug funds, and the participation of key stakeholders in decision making over the allocation of locally collected drug funds.

## Background

The shortage of drugs and medical supplies for maternal health is a challenge facing many health systems in low and middle income countries. This contributes to the provision of poor quality maternal health services and consequently to maternal deaths [1]. It is estimated that almost 99% of all maternal deaths occur in developing countries and these are mostly in women living in rural areas [2]. Most of these deaths could be avoided if women were given access to adequate maternal health services including quality emergency obstetric care.

Recently, the provision of quality emergency obstetric care has been advocated as a cost-effective intervention for reducing maternal deaths [3-5]. The concept of emergency obstetric care (EmOC) is based on the assumption that maternal complications are unpredictable and that obstetric complications can occur in around 15% of deliveries [6,7]. When such complications occur, maternal mortality could be prevented in a setting where skilled health attendants, drugs and medical supplies needed for EmOC are available [8].

Despite its importance in the provision of quality EmOC, the shortage of drugs and medical supplies is not commonly mentioned by policy makers or researchers when addressing the causes of poor maternal health services. Researches on quality EmOC have mostly addressed the need for skilled health attendants and for the referral system to provide quality EmOC [9-13]. Furthermore, the provision of obstetric care coverage has been evaluated against the presence of physical infrastructure such as number of health centers, without taking into account the actual care provided at these facilities [14,15]. This could lead to the perception that the coverage of EmOC had improved theoretically, while in practice the accessibility and utilization of these facilities is limited due to shortage of drugs and medical supplies. Previous studies that have reported on drug shortages in developing countries have focused mostly on diseases such as malaria, tuberculosis and HIV/AIDS [16-18], and very few studies have described the effects of drug availability on maternal health care [19-21].

There is a need for improved access to maternal health drugs and supplies, and challenges within the health systems that prevent access to these supplies have to be addressed. This study aims to explore the experiences of rural health facility managers in Tanzania on issues related to timely availabilities of drugs and medical supplies for EmOC in order to provide more understanding of the problem and identify potential areas for improvement.

### Drug delivery system within the decentralized health system in Tanzania

As a result of decentralization which took place in Tanzania in early 1990s, the local government authorities (LGAs) became responsible for resourcing the district health facilities (district hospitals, health centres, dispensaries) [22]. However the Ministry of Health and Social Welfare (MoH&SW) remained responsible for providing funds for drugs and medical supplies. These funds are disbursed to the local health facilities through the Medical Store Department (MSD), an organ of the Ministry responsible for managing procurement and supply of drugs and medical equipment [23-25]. Other source of funds for drugs and medical supplies is a fund collected as health insurance scheme from the community members known as Community Health Fund (CHF).

The ordering of drugs and medical supplies is done quarterly using a system known as Integrated Logistic System (ILS). Using the ILS, each facility manager can order drugs and medical supplies according to facility’s needs, through the District Medical Officer’s (DMO) office to the zonal MSD, which in turn distributes facility-specific packs directly to the facilities (see Table 1).

**Table 1:** Process of reporting and ordering of drugs and medical supplies using ILS

1.	Facility manager (dispensary/health centres) completes an order form for drugs and medical supplies known as RR form (Report & Request form).
2.	The completed form is then submitted to the district, reviewed by district pharmacist and approved by the DMO.
3.	The DMO then sends the drug order to the zonal MSD.
4.	The zonal MSD receives orders, prepare package of drugs and medical supplies as per the facility order and seals each order in cartons.
5.	The sealed cartons are transported directly from zonal MSD to the facility level.
6.	Upon arrival at the facility, cartons are opened at the facility in the presence of one member of the health facility governing committee, and then counted and entered in the store ledger.

The ILS, which replaced the former kit system in the mid-2000s, was expected to improve the drug availability at health facilities by minimizing the supply of drugs which are not used frequently. Through the routine reporting of data coupled with the routine ordering of resupplies, it was thought that the ILS would increase accountability and better inform the decision maker in the central government of the increased drug needs [26].Though this system was reported to have minimized the stock-out rate [26], its impact on improving the availability of drug and medical supplies has not yet been documented.

In Tanzania, drug and medical supply availability at health facilities remains uncertain. Some assessments and drug tracking studies have reported poor availability of drugs and medical supplies, with the facilities at the lower level of the health system being more affected [23,25,27]. The report of a National Health Service provision assessment undertaken in 2006 indicated that only one in eight facilities with delivery services had basic equipment and medical supplies for conducting normal deliveries [28]. A recent assessment conducted in four regions of Tanzania has found that oxytocin, an essential medicine in preventing and treating post-partum hemorrhage (PPH) was not available in most of the visited facilities in the Lindi and Mtwara regions [27].

The government through its strategy of reducing maternal deaths [29], is working towards strengthening all dispensaries and health centers to provide basic emergency obstetric care (BEmOC), including the administration of antibiotics, oxytocics and anticonvulsants; performing manual removal of placenta, removal of retained products following miscarriage or abortion and assisted vaginal delivery. The government is also working to strengthen all hospitals and upgrade 50% of health centers to provide comprehensive emergency obstetric care (CEmOC) which include in addition to the aforementioned, the provision of cesarean section and safe blood transfusion [29].

## Methods

### Study setting

The study was conducted in Kongwa, a typical rural district in Dodoma region which had a total population of about 309,973 people in 2012 [30]. The two main ethnic groups found in this district are Gogo and Kaguru. Common occupations include subsistence farming and small scale trading. The district has one hospital, four rural health centers and 32 dispensaries that conduct delivery services. Many health facilities are accessed by unpaved rural roads, which are not easily passable during the rainy seasons. All these facilities provide antenatal care (ANC) and offer delivery services. Caesarean sections are provided at one rural health center and at the district hospital.

### Data collection

Different methods were used to collect data for this study. These included in-depth interviews with facility health managers, a survey of selected heath facilities and a desk review of existing/available documents.

#### In depth interviews

In depth interviews guides were designed to collect the views of facility managers on their experience of ensuring the timely availability of drugs and medical supplies for EmOC. In April and May 2012 a total of 17 health facility managers were interviewed: 14 from the dispensaries and three from health centers. Furthermore, two members of the CHMT and one member of the CHSB were also interviewed on their experiences of the drug funding mechanism for the health facilities. The facilities where respondents came from were purposively selected from three wards (Mkoka, Mlali, Ugogoni) to reflect the geographical diversity of the study. Respondents were also selected purposively to include those involved in drug ordering and reporting using ILS. The two CHMT members, the District Medical Officer and District Pharmacist and a chairperson of the CHSB were interviewed because of their involvement in the approval of funds for drug procurement at the district level. The first author together with the research assistant collected data and all interviews were audio-taped; field notes and memos were written up both during and immediately after the interviews. The interviews followed a semi-structured format, and the following aspects were explored: availability of drugs and medical supplies and its effect on care, focusing on EmOC; the accessibility of complementary drug funds; and community and staff reactions to drug shortages.

#### Facility survey

During April 2012, 18 government-owned facilities were surveyed by the first author with the aim of understanding the existing situation with regard to the availability of drugs and supplies needed for EmOC. At each visited facility, observation and assessment were conducted by interviewing facility managers using a facility survey guide. The researcher interviewed facility managers about aspects of human resources, infrastructure, availability of drugs and medical supplies, and their experience of deliveries. The data collected during the interviews were validated by observation of the facility drug storage rooms and informal discussion with facility managers, staffs and user representatives at some of the facilities. In particular, the observation during the facility survey was focused on the presence or absence of essential drugs and medical supplies for EmOC.

### Data analysis

Audio-taped interviews were transcribed by the first author and translated from Swahili into English. The transcripts together with the expanded field notes were the main data used for analysis. To ensure familiarization with the data, multiple readings of the transcript and expanded notes were conducted and data were analyzed using thematic analysis [31]. Thematic questions were preselected and the parts of the text that referred to those questions were marked and coded. Similar codes considered pertinent to the preset research question were grouped to form subthemes and similar subthemes formed a theme. The emerged themes were then shared among other authors and a consensus was reached about the various themes and how they fitted together. Data from document reviews and health facility surveys were used as supporting information in clarifying the concepts that emerged during thematic analysis.

### Ethical considerations

This study was approved by the review body of Muhimbili University of Health and Allied Sciences (MUHAS). Permission to conduct the study in Kongwa district was given by Dodoma Regional Administrative Secretary (RAS). Written consent to visit facilities was obtained from the District Medical Officer’s (DMO) office, the District Executive Director’s (DED) office and the District Administrative Secretary’s (DAS) office. Informed consent to participate was obtained from the participants after assurances of anonymity and confidentiality were given.

## Results

### Facility survey and observation

#### Facility characteristics

The survey was carried out in 18 health facilities (1 hospital, 3 health centres, 14 dispensaries) from three divisions (Ugogoni, Mlali, Zoissa). All the facilities were government owned and providing antenatal and delivery services. Two of the health facilities offered caesarean delivery. Three health facilities were located in town; the rest (15 health facilities) were in remote areas.

#### Availability of drugs and medical supplies needed for EmOC

All health facilities surveyed reported having adequate delivery packs. However, there were shortages of some essential drugs and supplies needed for EmOC in most of facilities on the day of survey (Table 2). Oxytocin, which is used for bleeding control, was found in very few facilities (28%); surprisingly, ergometrine, which is now not recommended for bleeding control, was found to be present in seven (39%) facilities. Despite its importance in the initial stabilization of women who experience obstetric emergencies, IV infusions, Ringer lactate and normal saline were present in only half of the facilities.

**Table 2:** Availability of selected drugs and medical supplies for EmOC

**Drug and medical supplies**	**Number of facilities reporting availability N = 18**
***Drugs***	
Any antibiotic injection	4(22%)
Oxytocin injection	5(28%)
Ergometrine injection	7(39%)
Diazepam injection	12(67%)
***Medical supplies***	
Ringer lactate	9(50%)
Dextrose	9(50%)
Normal saline	9(50%)

### Experience of health facility managers

From the analysis of the interviews, three themes emerged which described the experience of health facility managers in ensuring the timely availability of drugs and supplies needed for EmOC. Table 3 presents the themes and related subthemes.

**Table 3:** Themes and subthemes related to health facility managers experience of ensuring timely availability of drugs and supplies needed for EmOC

**Theme**	**Subthemes**
Unreliability of getting drugs and medical supplies needed for EmOC	Long delay in supply of drug and medical supplies
	Supply of expired and unmatched drugs and medical supplies
	Denial of supply of needed drugs
Delivery of untimely and suboptimal EmOC services	Delay in delivering care to women
	Decreased trust in health workers
	Decreased morale of health workers
Under budgeting and limited accessibility to locally mobilized drug funds.	Insufficient funds from central government to meet local drug demand
	Bureaucratic process for accessing locally mobilized drug funds.

### Unreliability of getting drugs and supplies

#### Long delay in supply of drugs and medical supplies

Participants in this study reported difficulties in obtaining the drugs and medical supplies they needed on time for EmOC services at their facilities. Generally, the availability of drugs was reported to be challenging and the facility could be without drugs for a long time, as described by one respondent:

*Drug availability is a very difficult issue here. For the whole of the last quarter* [last three months]*, there were no drugs at the Centre. Drugs arrived just the day before yesterday. Even the nearby centers had no drugs. We depend on the district hospital to get drugs. When you request drugs today, they give you some, but when you go tomorrow you might be told that they don’t have them.* (Respondent 1)

Participants reported delays in getting ordered drugs and that no clear information was given by drug suppliers about the reasons for delay, leaving facility managers striving without knowing what to do, as expressed by one respondent:

*We have delays in getting drugs here. One time drugs were delayed, I asked the district pharmacist to tell me what happened, he told me to call the MSD people. The MSD people told me that they failed to bring drugs because the car had problems. This means the drugs were there but the problem was the car, and that is how things are.* (Respondent 1)

#### Supply of expired and unmatched drugs and medical supplies

It was also reported that the drugs and medical supplies brought to the facilities are sometimes expired or not those that were ordered.

*Sometime back, MSD people came here at 6.00 am hurried me to be ready to receive the kit; when we opened it, the kit included some expired drugs including egometrine, which is not used nowadays. I rejected the drugs.* (Respondent 2)

Moreover, drugs and medical supplies that were brought to the facilities were reported to be insufficient and not matched to the needs of the particular facility at that specified time.

*I ordered five boxes of gloves; they brought only two to be used for three months. In this dispensary we conduct deliveries daily, so I need more gloves. These two boxes I will just use up in one or two months, and you can open these boxes and find that some gloves are not good to use at all.* (Respondent 3)

Some supplies were reported to be brought to the facilities from MSD despite being not useful.

*They don’t bring important supplies that you ordered; they just bring what is not important like registry books, prescription forms, which are not that needed at the dispensary or health center and they still use the same money you have for the drugs. See how the system is wrecked.* (Respondent 3)

#### Denial of supply for needed drugs at dispensary level

Some participants argued that their facilities conducted a number of deliveries and sometimes experienced obstetric complications. However, they are denied access to certain types of drugs and hence fail to manage these complications once they occur. They further argued their need to be supplied with such drugs as they help to stabilize women before referring them to a higher level.

*You can be in need of certain drugs because of complications which sometimes occur at our facility, but you are not given them just because it is a dispensary. I am getting real emergency cases here. I had a woman who had fits. I had to give a magnesium sulphate injection before I referred her. I couldn’t because I did not have any. You have seen our roads, it’s about five to six hours from here to X health centre and they are not easily passable, especially during the rainy season. A woman can die while you are watching. It’s better to have two or three bottles for emergencies, because each year we can have one or two cases like this*. (Respondent 4)

### Delivery of untimely and suboptimal EmOC services

Participants in this study described several negative effects on EmOC services of the shortage of drugs and medical supplies in their facilities. The perceived effects included the following:

#### Delay of delivering care to women

Lack of drugs at the facility was mentioned as a cause of delay in the provision of services, especially when emergency obstetric situations occurred. It was reported that at one time a facility that conducted emergency caesarean sections did not have most of the drugs. Relatives were requested to go and buy some supplies such as infusion and parenteral antibiotics before the operation started. This could delay the provision of appropriate care and place post-operative mothers at risk by exposing them to the possibility of developing complication like sepsis.

Moreover, the lack of drugs and necessary medical supplies can compromise the quality of EmOC given to mothers. For instance, one respondent mentioned that oxytocin injection was given only to mothers with excessive bleeding and not given routinely to all mothers after delivery to prevent post-partum hemorrhage, as recommended in the guideline.

*I had no oxytocin here, I got five ampules from X dispensary. They are not enough, I don’t use it for all mothers as is recommended, but only for those with excessive bleeding, and when there is no oxytocin at all, I just pray that nothing happens*. (Respondent 5)

#### Lack of trust in health workers

Lack of drugs at the facility created a relationship of distrust between users and health care providers. It was reported that the community perceived health care providers as those who took the drugs and used them for their own purposes or sold them, causing a drugs shortage at the facility, as this respondent reported:

*When drugs are unavailable people complain; they perceive us differently. They are saying that, we, health care providers, are taking drugs from here and keeping them in our houses or that we open medical stores. This is because if they go to drug stores, drugs are available but there are none in the hospital; when drugs are out of stock, people misunderstand us.* (Respondent 7)

It was reported that lack of trust in health workers resulted in low utilization of facilities and a reduction in contribution to the Community Health Fund (CHF).

#### Decreased morale of health workers

Respondents reported having a hard time when the facility lacked drugs. Lack of drugs and medical supplies created a difficult working environment for health care providers. Respondents reported disappointment because they felt they were providing incomplete care, as this respondent reported:

*I feel very bad when drugs are not available. I used to struggle a lot, going to other health centers and dispensaries asking for drugs. Patients also suffer, especially those who underwent cesarean section. You can even become discouraged from continuing working. Sometimes you reach a point where you think that there is no point in continuing working and treating people.* (Respondent 8)

Furthermore, it was reported that lack of drugs exposed health workers to malpractice and poor work performance.

*When drugs go missing at the facility, it really becomes difficult. You have been taught this way, you practice differently.* (Respondent 7)

### Under-budgeting and limited accessibility to locally mobilized drug funds

#### Insufficient funds from central government to meet local drugs demands

CHMT and board member of CHSB reported that the government had provided funds for drugs and medical supplies for each of the facilities. These funds were reported to be kept in a zonal MSD account. Some facility managers mentioned that the allocation of funds for each facility is small and does not take into account the drug needs of each facility.

*The government sets a small budget for drugs. It is not enough to meet drug demand at each facility*. (Respondent 9)

There was a plea from some participants for the government to increase the budget for drugs and medical supplies due to the increased demands for services at their facilities.

*There is increased demand for services here. When service increases, the government should take note of it and find a way to increase drug budget immediately*. (Respondent 8)

Lack of sufficient drugs funding from the central government was described by participants as deterring expectations of improved drug availability with the newly implemented drug ordering system, ILS.

*Implementation of ILS is opposite to what we expected. We have been told that by using ILS we can order according to what we need. But now they put limits; you order as per your facility’s need, they tell you the budget is not enough. So ordering according to our needs is not there, though we do that on paper, but when drugs come a lot of items are missing just because the money you have at the MSD account is not enough. So ordering according to what we need with ILS is too theoretical*. (Respondent 9)

However, most participants reported being satisfied with the ILS system, and just mentioned the need for an accountability mechanism to be put in place to make the system work better.

*If auditors were in place, MSD would be accountable and responsible. Close follow-up of ILS, the drug delivery system, is needed so that the community can get good services. Without that no changes should be expected, because if there is no follow-up, things become worse*. (Respondent 10)

#### Bureaucratic process of accessing locally mobilized drug funds

Respondents reported other sources of funds for drugs which included Community Health Funds (CHF) and user fees which are collected locally and kept centrally in the district account. Though CHF was meant to act as complementary source of drug funds for a facility during a drugs shortage, the accessibility of funds was reported to be difficult due to its complicated approval process.

*“CHF money was supposed to be used for buying drugs during the drug crisis. We collect CHF from community members and keep it in the district account. But then to get them is a difficult process. We once wrote to get money but the district people delayed responding. You can write to request money today and you get it after a month, it really takes time”.* (Respondent 10)

Furthermore, the community members nor the facility managers have direct control over this fund, as expressed by one respondent:

*Drug funds that we have collected from people are available but they are not in our control. They are just kept centrally.* (Respondent 8)

It was also noted that health facility managers (HFM) and members of Health Facility Governing Committee (HFGC) were responsible for sensitizing community members to contribute to the CHF. Each facility was responsible for collecting CHF money from the community members within the area it served, an activity that was reported to be well done by most HFMs. However, it was reported that despite their involvement in collecting CHF there was no involvement by the district authority in deciding on how to utilize the money that had been collected, as one respondent said:

“*I don’t know up to now how much money had been collected from my village; it is all kept at the district. I have not been given any feedback or a report on how much CHF was collected and what has been done with the collected money”.* (Respondent 11)

## Discussion

This study found a number of governance-related issues that affect the timely availability of essential drugs for EmOC at rural health facilities. The study focuses on availability of EmOC drugs and supplies due to their life saving role during maternal complications and the scarce research on this. The findings however most likely apply also to other essential drugs and supplies in this setting as the procurement and distribution system used is the same.

The supply chains were reported to have many problems including delays in the supply of ordered drugs, the supply of unneeded drugs and medical supplies, and the supply of poor quality drugs. The lack of measures for holding accountable those who were irresponsible could have contributed to this problem. Other issues revealed by this study were the inaccessibility of alternative drug funds, the lack of transparency of fund utilization and the minimal involvement of facility managers and community members in decision making over locally mobilized drug funds. The same findings have also been reported in studies done elsewhere [19,25]. Though these are common health system challenges related to drugs and medical supplies [32,33], their existence will impair the provision of quality EmOC, especially in rural settings.

### Availability of drugs and medical supplies and provision of quality EmOC

The provision of quality EmOC not only depends on skilled personnel, but also on the availability of essential drugs and supplies for EmOC. Our study found that availability of essential drugs and medical supplies in rural health facilities is still a problem, a situation that could interfere with provision of quality EmOC at this level of health system [8,32,34-36]. As a result, the quality of care and adequacy of these facilities to provide essential EmOC services is questionable. Lack of drugs and medical supplies at the facilities means delays in early interventions in obstetric emergencies, which contributes to maternal deaths at health facility level [8,20,37]. This implies that the mere use of facilities by mothers for deliveries would not reduce maternal mortality automatically; rather, mortality is affected by the quality of services offered, which are determined by, amongst other things, the adequate availability of medical supplies and drugs. A recent study in India indicated that increased access to facility deliveries did not result in the reduction of maternal deaths because it was connected to a weak supply system that led facilities to provide poor quality obstetric care [34]. Other studies in Tanzania have also reported the provision of poor obstetric care due to lack of drugs and medical supplies at health facilities [38,39]. Furthermore, an inadequate supply of drugs and medical supplies results in a difficult working environment and causes a worsening of staff morale [19,38,39].

### Drug supply system and accountability measures to address long delays and the supply of unordered and expired drugs

To ensure drugs and medical supplies are made available in a timely way at health facilities, a functioning country-wide drug supply system should be in place. In addition, each player in the system should be responsible and accountable for making the system operate. This should be the same for the MSD if a drug supply at facility level is to be maintained. However, in this study the performance of the MSD was found to be suboptimal even with the use of the ILS, an ordering and reporting tool introduced to make the system more efficient. Lack of clear measures for holding irresponsible players to account within the distribution system was explained as constraining the better outcomes expected from ILS use. While the supply of expired drugs and medical supplies found in this study could have endangered users’ lives, no clear explanations were given or disciplinary actions taken against those who were responsible. Lack of accountability has contributed to the poor performance of many health systems in developing countries [40-43].

### Drug shortages and challenges in accessing locally mobilized drug funds

The drug funds set aside by the government for each facility through the MSD account were reported to be insufficient. As a result, the MSD failed to supply all the ordered drugs, resulting in a shortage of drugs at the facilities, including those needed for EmOC services. The Community Health Fund scheme, which was also implemented in other districts in Tanzania, was one strategy for generating an alternative drugs fund [44-46]. Despite the good response from the community to contributing into this scheme, access to these funds, which are kept centrally at the district account, has been difficult. The long approval process and the need for more than five signatories before securing access to the fund has prevented the scheme from being an urgent source of complementary funding during drugs crises at the facilities. Facilities continue to lack drugs, which reduces the community’s motivation to continue contributing due to loss of trust over the use of the money already contributed. The findings of this study suggest the need for the increased autonomy of health facilities over the CHF to ensure the sustainability of the scheme.

### Transparency and stakeholder’s involvement in decision making over the utilization of drug funds

In this study, transparency over the utilization of drug funds, both those from the central government and those locally mobilized through the CHF scheme was found to be very low. Furthermore, neither the facility managers, who were in the frontline of mobilizing community members to contribute to CHF, nor community representatives were found to be involved in decision making over the drug funds at the district level. These findings suggest that, despite the decentralization process that is going on in the health care sector in Tanzania, community and key stakeholder involvement in decision making is still a challenge. The lack of transparency and participatory decision making observed in this study could create opportunities for fund misuse. Community mistrust towards health managers has resulted in poor implementation of the CHF scheme in other areas of Tanzania [46-49].

### Methodological considerations and study limitations

This paper highlights the governance-related challenges that health facility managers encountered in ensuring the timely availability of drugs and medical supplies for EmOC. The study used a qualitative approach whose trustworthiness is assessed using four criteria: credibility, transferability, dependability and confirmability [50]. Most of the participants interviewed were both leaders and service providers from the different levels of the district health system; therefore, they provided in-depth information about the problem under study. To achieve credibility of the data, this information was triangulated with that obtained from the observations during the facility survey, the document review and expanded field notes. Records of research activities were kept carefully to enhance the follow-up of the data, hence the data were dependable. Despite the researcher’s work experience in a maternity ward as a midwife for some time, the analysis was based on data collected rather than on the researcher’s pre-existing understanding of the problem. This can be confirmed by the coding of the collected data and quotes that support the presented findings. Though the findings cannot be transferred directly into other contexts, they could reflectively mirror what is happening in other districts, taking the study setting as a case study.

Despite the usefulness of the study for providing enlightenment of how governance-related challenges affect the availability of essential drugs and medical supplies for EmOC, some limitations were observed. Suppliers’ perceptions of the problem were not explored, nor were the operational challenges faced by the drug distribution system. In this study, issues such as the long delays and the supply of expired drugs and unneeded medical supplies were reported by participants, suggesting the ineffective functioning of the distribution system, a problem found in other similar studies [20,21,39]. Involving participants from the supply system would provide a broader picture of the problem from different perspectives.

As noted in this study, members of the Health Facility Governing Committee (HFGC) were involved in an approval process for the drug fund that is locally mobilized and they are responsible for overseeing drug management at facility level. However, their perspectives as well as those of women were not explored in this study as the researchers wanted to focus on professionals’ perspectives of the problem. Nevertheless, studying their perspectives would have added a deeper understanding of the community involvement in decision making on the local health agenda. Furthermore, perspectives of women using these health facility would have added a different perspective, useful for triangulating the findings.

Observations conducted during the facility survey focused only on the availability of a few selected essential drugs and medical supplies needed for EmOC on the day of the survey. Neither the duration nor the extent of the problem was explored in this study. However, the researchers focused on the challenges of obtaining drugs and medical supplies for providing quality EmOC on time and not on the wider magnitude of the problems.

## Conclusion

Obtaining drugs and supplies for EmOC in rural health facilities is still unreliable, which compromises the timely provision of quality EmOC. Improving the governance of the delivery system in a way that promotes accountability and transparency in handling drug funds and also the participation of key stakeholders in decision making about the use of locally collected funds could improve the situation.

The provision of adequate and quality EmOC is vital for reducing maternal death and improving women’s health in general. However, this can be achieved if mothers have access to facilities with skilled attendants supported by adequate equipment, supplies and drugs. With the current policy of increasing institutional delivery in Tanzania, efforts should be made to ensure the quality of services provided, especially in rural areas. This should imply, amongst other things, that drugs and medical supplies are adequately available and accessible to women when they need them. However, adequate drug financing from the government and flexible and supportive drug policies should be in place to achieve this goal.

## Competing interests

The authors declare that they have no competing interests.

## Authors’ contributions

DAM conceived the study, participated in its design, carried out interviews and analysis, and drafted the manuscript. AK and MM participated in the design, were responsible for the overall coordination and helped to draft the manuscript. IG and AKH participated in the design and analysis and helped to draft the manuscript. All authors read and approved the final manuscript.

## Pre-publication history

The pre-publication history for this paper can be accessed here:

http://www.biomedcentral.com/1471-2393/14/108/prepub

## References

[B1] Yeager B Improving Access to Maternal Health Commodities: A System Approach 2012 http://maternalhealthtaskforce.org/discuss/wpblog

[B2] WHO Maternal mortality Factsheet 2012 http://www.who.int/mediacentre/factsheets/fs348/en/

[B3] Ronsmans C Kraham WJ Maternal mortality; who, when, where and why Lancet 2006 368 9542 1189 1200 10.1016/S0140-6736(06)69380-X 17011946

[B4] Jamison DT Feachem RG Makgoba MW Disease and Mortality in Sub-Saharan Africa 2006 2 Washington (DC): World Bank 21290641

[B5] Fournier P Dumont A Tourigny C Dunkley G Dramé S Improved access to comprehensive emergency obstetric care and its Effect on institutional maternal mortality in rur al Mali Bull World Health Organ 2009 87 30 38 d0i:10.2471/BLT.07.047078 10.2471/BLT.07.047076 19197402 PMC2649605

[B6] Cham M Sunby J Vangeri S Maternal mortality in the Rural Gambia, a qualitative study on access to emergency obstetric care Reprod Health 2005 2 1 3 10.1186/1742-4755-2-3 15871743 PMC1142340

[B7] World Health Organization Mother-baby package: implementing safe motherhood in countries 1994 Geneva: Maternal Health and Safe Motherhood Programme, Division of Family Health, WHO 88

[B8] UNFPA Emergency Obstetric Care Providing Emergency Obstetric and Newborn Care to All in Need http://www.unfpa.org/public/mothers/pid/4385

[B9] WHO Maternal and perinatal healthhttp://www.who.int/reproductivehealth/topics/maternal_perinatal/epidemiology/en/

[B10] Olsen OE Ndeki S Norheim OF Human resources for emergency obstetric care in Northern Tanzania; distribution of quality or quality? Hum Resour Health 2005 3 5 10.1186/1478-4491-3-5 16053519 PMC1199615

[B11] Pembe AB Carlstedt A Urassa DP Lindmark G Lennarth N Darj E Effectiveness of maternal referral system in a rural setting: a case study from Rufiji district, Tanzania BMC Health Serv Res 2010 10 326 10.1186/1472-6963-10-326 21129178 PMC3003655

[B12] Spangler SA Assessing skilled birth attendants and emergency obstetric care in rural Tanzania: the inadequacy of using global standards and indicators to measure local realities Reprod Health Matters 2012 20 39 133 141 10.1016/S0968-8080(12)39603-4 22789091

[B13] UNFPA Skilled Attendance at Birth http://www.unfpa.org/public/mothers/pid/4383

[B14] Echoka E Kombe Y Dubourg D Makokha A Olsen BE Mwangi M Byskov J Olsen QE Mutisya R Existence and functionality of emergency obstetric care services at district level in Kenya: theoretical coverage versus reality BMC Health Serv Res 2013 13 113 10.1186/1472-6963-13-113 23522087 PMC3616893

[B15] Prytherch H Massawe S Kuelker R Hunger C Mtatifikolo F Jahn A The unmet need for emergency obstetric care in Tanga Region, Tanzania BMC Pregnancy & Childbirth 2007 7 16 10.1186/1471-2393-7-16 17683590 PMC1988833

[B16] Towse A Keuffel E Keffler HE Ridley DB Drugs and vaccines for developing countries 2011 https://faculty.fuqua.duke.edu/

[B17] Tumwine Y Kutyabami P Odor AR Kalyango JN Availability and expiry of essential medicines and supplies during the “Pull” and “Push” Drug acquisition systems in rural Ugandan Hospital Trop J Pharmaceut Res 2010 9 6 557 564

[B18] Harding R Simms V Penfold S Downing J Powell RA Mwangi-Powell F Namisango E Moreland S Gikaara N Atieno M Kataike J Nsubuga C Munene G Banga G Higginson IJ Availability of essential drugs for managing HIV-related pain and symptoms within 120 PEPFAR-funded health facilities in East Africa: a cross section survey with onsite verification Palliat Med 2013 10.1177/0269216313498637 23885009

[B19] Penfold S Shamba D Hanson C Jaribu J Manzi F Marchant T Ramsey K Schellenberg D Schellenberg JA Staff experiences of providing maternity services in rural southern Tanzania- a focus on equipment, drug and supply issues BMC Health Serv Res 2013 13 61 10.1186/1472-6963-13-61 23410228 PMC3599073

[B20] Ziraba AK Mills S Madise N Saliku T Fotso J The state of emergency obstetric care services in Nairobi informal settlements and environs. Results from a maternity health facility survey BMC Health Serv Res 2009 9 46 10.1186/1472-6963-9-46 19284626 PMC2662828

[B21] Pearson L Shoo R Availability and use of emergency obstetric services: Kenya, Rwanda, Southern Sudan and Uganda Int J Gynecol Obstet 2005 88 208 215 10.1016/j.ijgo.2004.09.027 15694109

[B22] The Ministry of Health and Social Welfare Tanzania (MOHSW) Modular course in district health management 2007 Dar-es-Salaam, Tanzania http://www.tgpsh.or.tz/fileadmin/documents/JAST/Module_CM3.pdf

[B23] Euro Health Group and MSH Tanzania The United Republic of Tanzania. Drug tracking study 2007 http://hdptz.esealtd.com/fileadmin/documents/Key_Sector_Documents/Tanzania_Key_Health_Documents/Tanzania_Drug_tracking_study_final_report.pdf

[B24] Medical store department http://www.msd.or.tz/

[B25] SIKIKA Medicines and medical supplies availability report. Using absorbent gauze availability survey as an entry point. A case of 71 districts and 30 health facilities across mainland Tanzania 2011 http://www.sikika.or.tz/en/cms/functions/files/publication70.pdf

[B26] Tanzania Integrated logistics system pilot test evaluation. Using the logistics indicator assessment tool USAID 2005

[B27] Tanzanian German Programme to Support Health (TGPSH) Availability and management of medicines and medical supplies. Findings from an assessment of 87 health facilities in four Regions in Tanzania 2011 Dar es Salaam, Tanzania http://www.tgpsh.or.tz/fileadmin/documents/SHI_2/GIZ_Medicines_Management_Assessment_2011_final.pdf

[B28] National Bureau of Statistics (NBS) [Tanzania] and Macro International Inc Tanzania Service Provision Assessment Survey 2006 2007 Dar es Salaam, Tanzania http://dhsprogram.com/pubs/pdf/SPA12/SPA12.pdf

[B29] Ministry of Health and Social Welfare The National Road Map Strategic Plan To Accelerate Reduction of Maternal New-born and Child Deaths in Tanzania, 2008 – 2015 2008 http://www.unfpa.org/sowmy/resources/docs/library/R224_MOHTanzania_2008_Roadmap_MNCH.pdf

[B30] National Bureau of Statistics Tanzania national population and housing census 2002 http://nbs.go.tz/tnada/index.php/catalog/7

[B31] Braun V Clarke V Using thematic analysis in psychology Qual Res Psychol 2006 3 2 77 10 10.1191/1478088706qp063oa

[B32] Research report No. 1 Governing health service delivery in Uganda. A tracking study of drug delivery mechanisms http://ageconsearch.umn.edu/bitstream/97932/2/research_report1.pdf

[B33] van Olmen J Criel B van Damme W: Marchal B van Belle S van Dormael M Hoeree T Pirard M Kegels G Analysing Health Systems To Make Them Stronger. Studies in Health Services Organization & Policy 2010 Antwerp, Belgium: ITGPress, Nationalestraat 155, B-2000

[B34] Randive B Diwan V Costa AD Indian’s conditional cash transfer programme (The JYS) to promote institutional birth: is there an association between institutional birth proportion and maternal mortality? PLOS ONE 2013 8 6 e67452 10.1371/journal.pone.0067452 23826302 PMC3694862

[B35] Safe Motherhood Inter-Agency Group Technical Consultation “Ensure skilled attendance at delivery(2000a) (2000) Geneva: New York: SMIAG/FCI; 2000

[B36] Emergency Obstetric Care Quick Reference Guide for Frontline Providers. Emergency. Obstetric Care. United States Agency for. International Development [pdf.usaid.gov/pdf_docs/PNACY580.pdf]

[B37] MOHWS Emergency Obstetric Care Job Aid 2008 Dare-es-Salaam – Tanzania: Ministry of Health and Social Welfare

[B38] The Three Delays Modal. Maternity Worldwide Saving Lives in Childbirth http://www.maternityworldwide.org./wp-content/uploads/2010/07//Burare-22-wpc

[B39] Mselle LT Moland KM Mvungi A Olsen BE Kohi TW Why give birth in health facility?: Users’ and providers’ accounts of poor quality of birth care in Tanzania BMC Health Serv Res 2013 13 174 10.1186/1472-6963-13-174 23663299 PMC3654954

[B40] Olsen OE Ndeki S Norheim OF Availability, distribution and use of emergency obstetric care in northern Tanzania Health Policy Plan 2005 20 3 167 175 10.1093/heapol/czi022 15840632

[B41] Emanuel EJ Emanuel LL What is accountability in health care 1996 http://annls.org/article.aspx?articleid=709376 10.7326/0003-4819-124-2-199601150-00007 8533999

[B42] Brinkerhoff accountability and health systems: overview, framework and strategies 2003 http://www.who.int/management/partnerships/accountability

[B43] Carmargo CB Accountability for better healthcare provision: A framework and guidelines to define understand and assess accountability in health systems 2011 Basel, Switzerland: Basel Institute on Governance, Steinenring 60, 4051 http://www.baselgovernance.org/fileadmin/docs/publications/working_papers/10_Accountability_for_better_healthcare.pdf

[B44] Msuya JM Juting JP Astaw A Impacts of community health insurance schemes on health care povision in Rural Tanzania 2004

[B45] Kamuzora P Gilson L Factors influencing implementation of the Community Health Fund in Tanzania Health Policy Plan 2007 22 95 102 doi:10.1093/heapol/czm001 10.1093/heapol/czm001 17299023

[B46] Mtei G Mulligan JO Community health funds in Tanzania A Literature Review 2007

[B47] Chee G Kimberly S Kapinga A Musau S Assessment of the Community Health Fund in Hanang District, Tanzania. Technical Report No.15 2002 Bethesda, MD: Partnerships for Health Reform (PHR), Abt Associates Inc

[B48] Musau S The Community Health Fund: Assessing implementation of new management procedures in Hanang District, Tanzania. Technical Report No.34 2004 Bethesda, MD: Partnerships for Health Reform (PHR), Abt Associates Inc

[B49] United Republic of Tanzania (URT) Assessment of Community Health Fund in Tanzania: factors affecting enrolment and coverage 2003 Dar es Salaam: Ministry of Health

[B50] Granehein UH Lundman B Qualitative content analysis in nursing research: concepts, procedures and measures to achieve trustworthiness Nurse Educ Today 2004 24 105 112 10.1016/j.nedt.2003.10.001 14769454

